# Impact of the Tips From Former Smokers Campaign on Population-Level Smoking Cessation, 2012–2015

**DOI:** 10.5888/pcd15.180051

**Published:** 2018-05-31

**Authors:** Rebecca Murphy-Hoefer, Kevin C. Davis, Diane Beistle, Brian A. King, Jennifer Duke, Robert Rodes, Corinne Graffunder

**Affiliations:** 1 Centers for Disease Control and Prevention, Office on Smoking and Health, Atlanta, Georgia; 2 RTI International, Center for Health Policy Science and Tobacco Research, Research Triangle Park, North Carolina

## Abstract

This study provides estimates of the long-term cumulative impact of the Centers for Disease Control and Prevention’s national tobacco education campaign, Tips From Former Smokers (Tips), on population-level smoking cessation. We used recently published estimates of the association between increased Tips campaign media doses and quit attempts to calculate campaign-attributable population sustained (6-month) quits during 2012–2015. Tips led to approximately 522,000 sustained quits during 2012–2015. These findings indicate that the Tips campaign’s comprehensive approach to combining evidence-based messages with the promotion of cessation resources was successful in achieving substantial long-term cigarette cessation at the population level over multiple years.

## Objective

Tobacco use is the leading cause of preventable death, disease, and disability in the United States, killing more than 480,000 American adults annually (1). Mass media campaigns are an evidence-based intervention for promoting cigarette cessation (1). In 2012, the Centers for Disease Control and Prevention (CDC) launched the Tips From Former Smokers (Tips) mass media campaign, featuring former smokers living with serious health effects from smoking. Studies have demonstrated significant annual quit attempts and cigarette cessation (2–4); however, no study has assessed the campaign’s combined, multiyear impact. The objective of this study was to assess the cumulative impact of Tips on sustained (6-month) cigarette abstinence during the first 4 years of the campaign’s implementation (2012–2015).

## Methods

Published estimates of the association between Tips campaign gross ratings points (GRPs, a measure of market-level campaign dose) and increased quit attempts were used to calculate population-level sustained quits attributable to the campaign. During 2012–2015, the campaign delivered an average 1,100 quarterly GRPs, consistent with CDC’s *Best Practices for Comprehensive Tobacco Control Programs — 2014* (5). Davis and colleagues estimated that an average delivery of 1,000 Tips GRPs per quarter was associated with an average 4.3 absolute percentage-point increase in quit attempts among adult smokers (2); this estimate was based on an analysis of a nationally representative online panel of US smokers that was designed to detect the effect of 1,000 Tips GRPs on quit attempts in the previous 3 months at approximately 89% power using 2-sided testing (2).

We combined data on previously estimated campaign effect size with census data of the US adult population and National Health Interview Survey (NHIS) estimates of adult cigarette smoking prevalence to derive quarterly population totals of smokers who attempted to quit in association with each campaign phase. We then multiplied the estimated increase in quarterly population quit attempts associated with the campaign by the number of quarters the Tips campaign was on air. This yielded an estimate of adjusted total campaign-attributable quit attempts for each campaign phase.

To calculate campaign-attributable sustained quits, we used data from Neff and colleagues (4), who estimated that 5.7% of smokers who attempted to quit in the previous 3 months remained abstinent from cigarettes in 3 subsequent follow-up surveys spanning approximately 6 months. We chose this quit rate because it was derived from the same data used to estimate the primary Tips campaign effects (2). Thus, each estimate of additional campaign-attributable quit attempts was multiplied by 5.7% to yield total campaign-attributable sustained quits during 2012–2015. We calculated upper-bound and lower-bound estimates of campaign-attributable sustained quits based on the lower and upper ranges of CDC’s *Best Practices* recommendations for quarterly dosing of tobacco education advertising (800 and 1,200 GRPs, respectively) (5).

## Results

During 2012–2015, the Tips campaign was associated with approximately 9.15 million total additional quit attempts among the US smoker population (Table). Based on the assumed 5.7% cigarette abstinence rate among quit attempters, this translates into approximately 522,000 campaign-attributable sustained quits during 2012–2015.

**Table:** Projected Campaign-Attributable Sustained Quits by Campaign Year for the Tips From Former Smokers Campaign, 2012–2015

Campaign Year	Dates on Air	Total No. of Quarters on Air	Adult Population Aged ≥18^a^	Current Cigarette Smoking Among Adults Aged ≥18, %^b^	No. of Adult Cigarette Smokers^c^	Total No. of GRPs Delivered^d^	No. of GRPs Delivered Per Quarter^e^	Average No. of Additional Quit Attempts Per Quarter^f^	Estimated Total No. of Additional Quit Attempts^g^	Estimated Total No. of Sustained Quits^h^
2012	Mar 19–Jun 19	1.00	240,291,024	18.1	43,492,675	1,040	1,040	1,870,000	1,870,000	107,000
2013	Mar 4–Jun 17	1.16	242,625,484	17.9	43,429,962	1,237	1,066	1,867,000	2,166,000	123,000
2014 (Phase 1)	Feb 3–Apr 6	0.75	244,986,302	17.0	41,647,671	861	1,148	1,791,000	1,343,000	77,000
2014 (Phase 2)	Jul 7–Sep 7	0.75	244,986,302	17.0	41,647,671	834	1,112	1,791,000	1,343,000	77,000
2015	Mar 30–Aug 16	1.49	247,279,859	15.3	37,833,818	1,890	1,268	1,627,000	2,424,000	138,000

Abbreviation: GRP, gross rating point.

a Data source: US Census Bureau, Population Division (6).

b Data source: Clark et al (7).

c Adult population multiplied by prevalence of cigarette use. Mean = 41,610,360.

d GRPs are derived from national population-weighted total GRPs delivered from campaign GRP reports.

e Total quarters on air multiplied by total GRPs delivered. Mean = 1,127.

f Adult smoker population size multiplied by 4.3% (average quarterly increase in past 3-month quit attempts per 1,000 GRPs).

g Total quarters on air multiplied by average additional quit attempts per quarter. Total = 9,146,000.

h Total additional quit attempts multiplied by 5.7% (estimated sustained quit rate among quit attempters). Total = 522,000.

The campaign would have generated a minimum of approximately 461,000 and a maximum of roughly 571,000 sustained quits had the campaign been delivered at the lower and upper bounds of CDC-recommended media dosing (800 and 1,200 quarterly GRPs, respectively).

## Discussion

Our study found that CDC’s Tips campaign was associated with more than a half-million sustained quits among US adult smokers during 2012–2015. These findings indicate that the Tips campaign’s comprehensive approach to combining evidence-based messages with the promotion of cessation resources, such as 1–800-QUIT-NOW quitline information and other resources on the campaign’s website, was successful in achieving substantial long-term cigarette cessation at the population level over multiple years. These findings are consistent with the growing body of scientific literature demonstrating the Tips campaign’s impact on increased calls to quitlines (8,9), knowledge of tobacco-related health risks (10,11), ability to serve various groups (ie, groups that differ by race/ethnicity, educational attainment, and mental health status) equally well (2), and cessation-related information seeking, including visits to the campaign’s website (12). Taken as a whole, these studies reinforce the importance of sustained, evidence-based mass-reach public education cessation campaigns as an effective way of encouraging smokers to quit (1).

The estimates of campaign-attributable sustained quits in this study are likely conservative given the use of a previously published effect size of a 4.3 percentage-point increase in quit attempts per 1,000 quarterly campaign GRPs (2). The actual quarterly delivery of the campaign was slightly higher during its implementation (approximately 1,100 GRPs per quarter), but we found no published effect size for this dose level. In addition, a relatively conservative, but recent, estimate of sustained quitting (5.7%) was used (4), although previous research found higher long-term cigarette abstinence rates (up to 9.0%) from tobacco control policy or clinical interventions (13).

This analysis has several limitations. First, it relies on a single overall average campaign effect estimated during the campaign timeframe (2012–2015). The actual campaign effect may have varied across years, although previous research did not find significant evidence of variation in effects by year (2). Second, the estimated sustained quit-rate measures sustained quitting over a 6-month period; it is possible that a percentage of sustained quitters may still relapse. Third, the study captured only the effects of the television component of the campaign, not other smaller campaign components such as billboards, radio, and digital advertising (2); thus, the estimated campaign effect size would be expected to be greater if these other components were included.

Our study is the first to place the existing scientific evidence base of CDC’s Tips campaign’s effectiveness in a broader context of multiyear population impact on smoking cessation. Our findings indicate that the campaign may have motivated more than a half-million successful quits among smokers, thus contributing to the decline in US adult cigarette smoking observed during this period (14). This finding underscores the critical role of national tobacco education campaigns as a counterpoint to the substantial pro-tobacco advertising and promotion that persists in the United States (1). Evidence of the effectiveness of the Tips campaign over multiple years reinforces the importance of continued use of evidence-based, national tobacco cessation media campaigns to further reduce tobacco use and tobacco-related disease and death.
